# Demystify False Dilemmas to Speak About Corruption in Health Systems: Different Actors, Different Perspectives, Different Strategies: Comment on "We Need to Talk About Corruption in Health Systems"

**DOI:** 10.15171/ijhpm.2019.61

**Published:** 2019-07-24

**Authors:** Karen Hussmann

**Affiliations:** Transparency International (TI), Berlin, Germany.

**Keywords:** Corruption in Health, Anti-corruption in Health, Corruption Risk Assessments, Conflicts of Interest

## Abstract

The call of the editorial of the International Journal of Health Policy and Management regarding the "Need to talk about corruption in health systems" is spot on. However, the perceived difficulties of why this is so should be explored from an actor’s perspective, as they differ for government actors, donors and the research community. In particular, false dilemmas around definition problems should be demystified, including by building systematic bridges between the anti-corruption/integrity and health policy communities of practice. In addition, the focus on corruption in frontline health service delivery generating mainly problems of access to health, needs to be complemented with addressing sophisticated kickback schemes, nepotism, and state capture of legislative and regulatory agencies and processes draining the health systems of large amounts of resources leading to another false dilemma of assumed sector underfunding. In terms of what can be done, comprehensive corruption experience and risk assessments conducted by independent actors, eg, universities, aimed at generating some basic consensus among the different actors of priority areas to be addressed on the basis of a co-responsibility approach could provide the basis for reform. Finally, governments and private sector actors in countries characterized by systemic corruption and clientelistic political systems will not reform themselves without strong and sustained demand from civil society and the media.

The call of the editorial of the International Journal of Health Policy and Management regarding the “Need to talk about corruption in health systems” is spot on.^1^ As explained in the editorial, corruption in health systems costs lives, not only by impeding access to life-saving treatments but also by killing people through the use of counterfeit or sub-standard drugs, eg, it impedes equitable access to quality care, it violates basic human rights, it undermines state legitimacy and trust in the health sector (both public and private), and it contributes to a possibly false dilemma of an assumed underfunding of the health sector. But there are estimates that allow to launch the hypothesis that if corruption in its multiple forms in the health sector was controlled, large part of the supposed underfunding could or would be resolved “Globally, over 7% of healthcare expenditure is lost to corruption. With annual global health expenditures now exceeding US$7.5 trillion, this suggests that far over US$500 billion in health resources are lost to corruption worldwide every year. Thus, curbing corruption in the sector could free up enough resources to pay for universal health coverage worldwide.”^2^

It is indeed surprising that despite an ever increasing body of evidence of the problem of corruption in health systems around the world – as revealed in international and a myriad of national perception surveys, corruption reports registered through the Anti-corruption Legal Advice Centers of Transparency International,^3,4^ incessant media reports on corruption scandals all over the world, including in relatively authoritarian regimes,^5^ a growing academic literature on corruption in the health sector, and persistent and publically reported corruption cases affecting the multimillion donor health programmes (bilateral, multilateral as well as vertical programmes) – the health policy community continues to have difficulties in many contexts to talk about corruption in health systems openly, frankly and with no fear of repercussions.

Given the multiplicity of actors involved in the health sector, as well as their different legitimate institutional roles, dynamics and (dis-)incentives, it might be useful to distinguish the exploration for the reasons of why this is so according to the nature of the different actors,^6^ and I will do the same for the suggestions of potential remedies. Some of the reasons may apply to several actors but others will influence the behavior of one particular actor while not the other. For example, in many countries it is difficult to use the term “corruption” as such in discussions with public sector officials or the political leadership. The term is often associated with gross crimes and nobody wants to be “titled” as being corrupt, which is often synonymous to being a criminal, and much less so when the corrupt practices in question err on the side of unethical behavior or administrative misconduct. On the other hand, for academic research it is usually not a great problem to use the term “corruption.” But for academics the definition problems might play a role when it comes to establishing a clear scope and focus of the research in question. Another example: the issues of “how do we conduct research on corruption in ways that capture what is really happening?” or “is it even legitimate to study corruption?” seem to be an impediment for researchers to talk about corruption. For government actors in-country one potential impediment lies in the fact that the institutional responsibility to develop, coordinate, and monitor anti-corruption policies usually lie with specialized central-government bodies, such as anti-corruption agencies, transparency or integrity commissions who manage the terminology and definitions of corruption,^7^ while health sector institutions tend to speak about irregularities and inefficiencies in particular. And on the side of donor agencies, reasons why there is little discussion about corruption in the health sector might include the perceived dilemma that open talks can have negative repercussions in the willingness to continue to invest in the sector (an aspect has been experienced by the author and colleagues in numerous occasions of anti-corruption on the health sector in countries from Afghanistan, through Cambodia, Ecuador, South Sudan to Zambia). Or in case that their sector counterparts do not address the issue in political and technical dialogues, donors tend to be reticent in pushing the issue given its political connotations and the fear of losing or damaging relationships that are crucial for programme implementation.

In addition to the before, it would be useful to explore the perceived difficulties around the definition of corruption in order to demystify some of them, something which might be achieved by building more systematic bridges between the anti-corruption/integrity and health policy communities of practice. In this sense, it is important to highlight that corruption encompasses a broad range of practices spanning from unethical behaviors, through administrative misdemeanors to outright criminal offences. In most countries, there is broad acceptance of what constitutes criminal offences and mostly administrative misconduct. Kickback schemes in procurement, insurance fraud, embezzlement of funds and diversion of budget transfers from one administrative level to another, position buying and selling, stealing of equipment, ambulances, etc are straight forward administrative, fiscal and/or penal crimes and therefore should not constitute definitional problems to talk about. On the other hand, ethical misconduct and behaviors that might be widely accepted although they are against the law, such as informal payments, absenteeism, etc are issues that require context specific analysis, discussion and agreement on how to deal with them, both from the policy and management perspective. Although all of these behaviors would classify as corruption, and do so in many countries, it is not the same to label a nurse or medical doctor involved in these types of “frontline” corruption with the same “corruption stigma” as the director of procurement of the national drug store who might be siphoning off hundreds of thousands of dollars through fraudulent drug procurement. Hence, one challenge to promote open discussion about corruption in the health sector is to develop clear language or terminologies on the different forms of corruption: from a research, from a public policy and from a public management perspective acknowledging the differences of impact, while avoiding to fall into an unhealthy “corruption relativism.”

Finally, there are a few enigmas in the health policy community when it does address corruption: on the one hand, the spotlight seems to be mainly shed on what can is often called “petty corruption” or corruption in frontline health service delivery, including informal payments, absenteeism, dual practice, inducement for unnecessary treatment, and there is a relatively broad body of literature on these issues. However, the types of corruption like sophisticated kickback schemes, serious conflicts of interest of high-level policy, regulatory and management officials, nepotism, and state capture of legislative and regulatory agencies and processes does not seem to get similar attention.^8^ While these types of corruption are more difficult to be captured in opinion and experience surveys, it is less understandable why there is relatively little documented experience or research although these, often criminal, forms of corruption have an enormous impact on the overall health system functioning as well as the available financial resources.^9^

Now, the question of what can or should be done, or which strategies can be pursued to get the different actors to talk about corruption in health systems: Similar to identifying the reasons of why there is a considerable silence on the issues, it would be useful to define different strategies for different actors taking into account their roles, dynamics, incentives as well as constraints.

Thus at country-level, in an ideal case scenario a comprehensive health sector corruption assessment would be carried out, focusing on both concrete experiences (where possible) as well as risks of corruption and would encompass the national level as well as a selection of sub-national level regions.^10^ Such an assessment would ideally be conducted by a well-respected university under the auspices of but independent from the health sector lead authorities (Minister of Health, Director of Health Insurance, Director of Superintendency, Director of Drug and Food Regulatory Body, etc). This first step of producing the necessary evidence is an excellent opportunity for donor support as many countries struggle to assign the necessary budget to such an activity. The results will provide inputs to convene key stakeholders of the whole system, discuss openly the problems and define jointly solutions. This generation of a political consensus in a particular country for the definition of priorities must include not only public sector actors but also those from the private sector, be it pharmaceutical or medical device companies, healthcare providers or private insurers.^11^ The definition of a concrete road map to address the different prioritized problems is crucial and will require context specific analysis of political economy in order to navigate with and through the powerful interest. An ensuing action plan with objectives should reflect ideally progress related with sector goals and to the extent possible health outcomes, while some indicators on increased access to information or accountability or the management of conflicts of interest, eg, might be useful complementary monitoring elements. It might be useful to foster inter-institutional coordination between health sector public entities and the respective anti-corruption or transparency body in order to feed into the national cross-cutting anti-corruption policies and public discourse. The latter could be particularly useful if there were additional elements of traction, such as international commitments, eg, the Open Government Partnership,^12^ the Public Contracting Partnership, regional or international conventions, the Sustainable Development Goals, etc.

With regard to the research community, in addition to the important suggestions of the editorial to promote inter-disciplinary research, academia can play an role to provide more insight on what works, what does not and under what conditions from perspective of more comparative research.^13^ Although corruption and in particular the mitigating measures are highly context specific, comparative research could help to offer inspirations, avoid pitfalls and prevent the need for reinventing the wheel. In addition, the research community could make an extremely relevant contribution to the field by producing more research on the “higher level” types of corruption, including policy and regulatory capture, conflicts of interest in high decision-making positions, the pharmaceutical value chain as well as their system-wide impact in terms of access to health but in particular on the financial sustainability of the system. And finally, the research community could support the health policy community by producing political economy and drivers of change analysis focused on the health sector but embedding this in the broader country context.

As for the donor community, despite increasing recognition of the problems and certain action taken to address them, there is ample room for improvement. The health and governance/anti-corruption units and experts of each donor should interact much more systematically both at headquarters and in country; inter-donor coordination in the field around corruption and anti-corruption approaches should be more explicit and at least minimally resourced; donors should provide regular support for the generation of country-level analysis to help provide inputs for rational national debates and policy-making. And in addition to supporting public sector reform, donors should provide funding, technical and “political” support to civil society organizations in view of strengthening demand for and monitoring of transparency and integrity approaches. The recently started initiative by the World Health Organization, United Nations Development Programme, and the Global Fund to create a Global Network on Anti-corruption, Transparency and accountability is a further and promising step to contribute to generating much needed evidence and coordination.^14^

Finally, governments and private sector actors in countries characterized by systemic corruption and clientelistic political systems will not reform themselves without strong and sustained demand from civil society and the media. If transformational change is to be achieved, it is key to strengthen these actors, build bridges and alliances between specific health sector organizations and governance or integrity promoting civil society organizations, work with journalists and media companies and develop strategies that will allow to sustain demand for reform through government changes as well as help avoid backsliding on progress made.

As stated in the editorial, a wider conversation about corruption in the health sector and how to address it is direly needed if universal healthcare coverage is to be achieved.

## Ethical issues

Not applicable.

## Competing interests

Author declares that she has no competing interests.

## Author’s contribution

KH is the single author of the paper.
